# An Evolutionary Framework for Understanding the Origin of Eukaryotes

**DOI:** 10.3390/biology5020018

**Published:** 2016-04-27

**Authors:** Neil W. Blackstone

**Affiliations:** Department of Biological Sciences, Northern Illinois University, DeKalb, IL 60115, USA; neilb@niu.edu; Tel.: +1-815-753-7899; Fax: +1-815-753-0461

**Keywords:** comparative method, eukaryotes, evolutionary theory, levels of selection, mitochondria, modern synthesis, phylogenetic systematics

## Abstract

Two major obstacles hinder the application of evolutionary theory to the origin of eukaryotes. The first is more apparent than real—the endosymbiosis that led to the mitochondrion is often described as “non-Darwinian” because it deviates from the incremental evolution championed by the modern synthesis. Nevertheless, endosymbiosis can be accommodated by a multi-level generalization of evolutionary theory, which Darwin himself pioneered. The second obstacle is more serious—all of the major features of eukaryotes were likely present in the last eukaryotic common ancestor thus rendering comparative methods ineffective. In addition to a multi-level theory, the development of rigorous, sequence-based phylogenetic and comparative methods represents the greatest achievement of modern evolutionary theory. Nevertheless, the rapid evolution of major features in the eukaryotic stem group requires the consideration of an alternative framework. Such a framework, based on the contingent nature of these evolutionary events, is developed and illustrated with three examples: the putative intron proliferation leading to the nucleus and the cell cycle; conflict and cooperation in the origin of eukaryotic bioenergetics; and the inter-relationship between aerobic metabolism, sterol synthesis, membranes, and sex. The modern synthesis thus provides sufficient scope to develop an evolutionary framework to understand the origin of eukaryotes.

“When all are one, and one is all,” *Stairway to Heaven*, Led Zeppelin

## 1. Introduction

In 1859, Darwin published the *Origin of Species*, in which he outlined his theory of evolution [1]. He perhaps most succinctly articulated his theory in the introductory lines of a later book, the *Descent of Man*, published in 1871: “He who wishes to decide whether man is the modified descendent of some pre-existing form, would probably first enquire whether man varies, however slightly, in bodily structure and in mental faculties; and if so, whether variations are transmitted to his offspring in accordance with the laws which prevail with the lower animals… The enquirer would next come to the important point, whether man tends to increase at so rapid a rate, as to lead to occasional severe struggles for existence; and consequently to beneficial variants, whether in body or mind, being preserved, and injurious ones eliminated” [2]. In short, according to Darwin, evolution occurred when heritable variation was subject to natural selection.

In the *Origin of Species*, Darwin’s stated goal was to demonstrate that the theory of “special creation”—that each species was created separately by God—was unnecessary. In this regard, he largely succeeded. Nevertheless, his theory of evolution by natural selection had few adherents. Where did variation come from? How was it inherited? In later editions of the *Origin*, Darwin invented increasingly fanciful answers to these questions. It was not until Mendel’s work was rediscovered in the early 20th century that one class of mechanisms of variation and inheritance began to be elucidated by the science of genetics. The modern synthesis of the 1930s and 1940s unified Darwin’s theory and Mendelian genetics [3].

The goal of this review is not to enumerate the many and various challenges to the modern synthesis that have developed in the intervening years. Rather, here I will focus on a particular challenge—the endosymbiotic theory of the origin of mitochondria. The endosymbiotic theory poses a long-recognized challenge to the modern synthesis: how can cooperation evolve if Darwinian competition is the driving forces of evolution? A trenchant examination of the nature of Darwin’s theory and of cooperation itself, however, suggests that this challenge is more apparent than real.

In addition, the endosymbiotic theory poses another challenge to the modern synthesis that has only recently become apparent: how can the evolution of eukaryotes be understood if their last common ancestor exhibited all of their derived features? In the only illustration in the *Origin of Species*, Darwin carried his theory to its obvious conclusions and sketched the first phylogenetic tree. Today, such trees are typically built with nucleotide sequence data using statistical and mathematical theory. Coupled with the comparative method [4], these trees allow reconstructing the sequence of character state changes in a succession of common ancestors. Yet the springing forth of the common ancestor of eukaryotes, fully formed from prokaryotic predecessors, confounds this approach. Here these issues will be examined more closely, and a possible way forward will be developed and illustrated with several examples.

## 2. Conflict and Cooperation in the Darwinian Evolution of Endosymbiosis

Early 20th century formulations of the endosymbiont theory of the origin of eukaryotes explicitly rejected “Darwinian” notions of conflict and posed cooperation as an alternative. For instance, Wallin [5] described “symbionticism” as a missing part of Darwin’s theory, seemingly outside the realm of natural selection: “Modern writers have recognized the insufficiency of Darwin’s hypothesis to explain the origin of species. The ‘unknown factor’ in organic evolution has been especially emphasized by Osborne, Bateson, Kellog, and other recent writers. This “unknown factor” is especially concerned with the origin of species.” [5]. According to Wallin, the unknown factor was what he termed symbionticism. In resurrecting the endosymbiont theory later in the 20th century, Margulis also had little to say about potential conflicts, despite discussing scenarios in which evolutionary conflict would seem inevitable: “Next, the view of evolution as chronic bloody competition among individuals and species, a popular distortion of Darwin’s notion of ‘survival of the fittest,’ dissolves before a new view of continual cooperation, strong interaction, and mutual dependence among life forms. Life did not take over the globe by combat, but by networking. Life forms multiplied and complexified by co-opting others, not by killing them.” [6].

General considerations of the evolution of cooperation have a long and tortured history [7,8]. Modern synthesis architects sometimes used sloppy language in discussions of selection, e.g., presenting selection as a force that acts for the benefit of the species. Potential misunderstandings became considerably more explicit with the work of Wynne-Edwards [9], who famously argued that species could be selected to limit their population sizes so as not to over-exploit their food resources. A number of evolutionary biologists, most notably Maynard Smith [10] and Williams [11], pointed out the obvious limitations of this argument: if some individuals reproduce indiscriminately, while the rest of the population limits its reproduction, the prolific individuals will leave more offspring. If this behavior is inherited, at least in part, it will increase in frequency in the population, even if this leads to the destruction of habitat and the eventual demise of the species. In some circumstances, the high rate of selection at the level of the individual can thus overpower the slower rate of selection at the level of the species. Of course, the discussion of Wynne-Edwards’ work suggests parallels to other examples of evolutionary conflicts at lower levels of the biological hierarchy, e.g., the evolution of cancer and the evolutionary interactions within a eukaryotic cell discussed in Section 4.2 below.

Nevertheless, selection can favor cooperation in several related ways and thus limit selfish replication. For instance, Hamilton [12] developed the idea that kin selection diminishes conflict and promotes cooperation among family members. Some regard kin selection as a special case of group selection, although this was not Hamilton’s view. If selection at the group level favors cooperative behavior, groups of selfish individuals will replicate slowly, and selfish behavior will be limited [13]. Indeed, Wilson and Wilson [14] trace this framework back to Darwin’s writings: “It must not be forgotten that although a high standard of morality gives but a slight or no advantage to each individual man and his children over the other men of the same tribe, yet that an increase in the number of well-endowed men and an advancement in the standard of morality will certainly give an immense advantage to one tribe over another. A tribe including many members who, from possessing in a high degree the spirit of patriotism, fidelity, obedience, courage, and sympathy, were always ready to aid one another, and to sacrifice themselves for the common good, would be victorious over most other tribes; and this would be natural selection. At all times throughout the world tribes have supplanted other tribes; and as morality is one important element in their success, the standard of morality and the number of well-endowed men will thus everywhere tend to rise and increase” [2].

In this passage, Darwin focuses on a trait—morality—that is assumed to be inherited at least in part and that “… gives but a slight or no advantage…” at the level of the human individual. In other words, at this level of the biological hierarchy, morality is selectively neutral. When individual-level selection alone operates, moral individuals will on average have no more offspring than immoral ones. Thus the frequency of moral individuals will neither increase nor decrease. Darwin then points out that at a higher biological level—the tribe—the results of selection are quite different: “A tribe including many members who, from possessing in a high degree the spirit of patriotism, fidelity, obedience, courage, and sympathy…would be victorious over most other tribes…”. In other words, when between-tribe conflict occurs, tribes that contain many moral individuals will prevail over tribes with fewer such individuals. Tribes that in aggregate have a high moral standard will increase in frequency relative to tribes that in aggregate have a low moral standard. The effects of tribe-level selection thus differ from the effects of individual-level selection. The latter will not affect the frequency of individuals that vary in moral standard, while the former very clearly does affect the frequency of tribes that in aggregate vary in moral standard. If between-tribe selection was a potent force in human evolution, the existence of human morality can be explained by this sort of natural selection.

**Figure 1:**
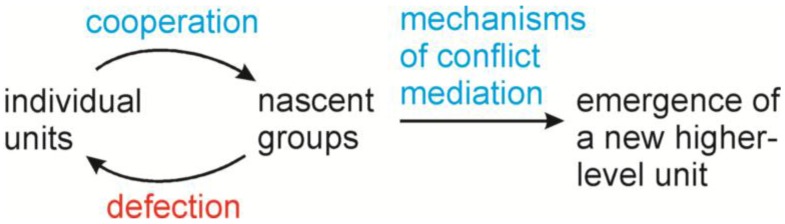
Cycles of cooperation and conflict occur repeatedly in the history of life with individual biological units banding together to form groups and defecting individuals weakening the integrity of groups. For a higher-level biological unit to form, mechanisms of conflict mediation must be derived [20].

In Darwin’s example, tribe-level selection may well encompass both kin and group selection. Kin and group selection often work together in biological systems, although adherents of the two explanations tend to disagree about this and much else besides [15]. Nevertheless, in the late 20th century a number of important works focused on the application of kin and group selection to the history of life [16,17,18]. Consider that staunch defenders of the primacy of individual-level selection typically worked with multicellular organisms. From the perspective of the history of life, however, what is a multicellular organism, but a group of cells? This sort of thinking decisively shifted the debate regarding levels of selection [8]. It is now generally accepted that repeatedly throughout the history of life, individual biological units banded together to form groups, driven at least in part by unceasing selection for size increase [19]. Clearly, selection on groups, including kin groups, had a major effect on increasing complexity in the history of life. Emerging complexity, however, was not automatic. The banding together of units into groups produced conflicts. These conflicts had to be mediated for the higher-level units to emerge (Figure 1). Thus conflict mediation was a crucial aspect of the emergence of complexity [20].

Early attempts to apply this framework to the mitochondrial endosymbiosis were largely ignored [21,22]. Rather, the recognition of conflictual stages in the early evolution of eukaryotes grew out of empirical findings that showed a role for mitochondria in programmed cell death [23,24,25]. In general, the mitochondrial endosymbiosis now fits comfortably within the multi-level theory of evolution. Mitochondrial ancestors and hosts banded together into nascent groups. In many of these groups, conflict overpowered cooperation and the lower-level units returned to the free-living state. Yet in one lineage, the group derived mechanisms of conflict mediation, and a new higher-level unit—the eukaryote—emerged. These mechanisms of conflict mediation likely constitute many of the shared derived features of eukaryotes [26,27,28,29,30,31].

## 3. Comparative Methods and the Origin of Eukaryotes

Architects of the modern synthesis tended to defer to “expert opinion” as the basis for determining the relationships among taxa. In the 1960s, two disciplines advocated more rigorous and quantitative approaches to phylogeny reconstruction. One group, the pheneticists, employed computer-based methods to examine as many characters as possible. The other group, the cladists, argued that only shared derived characters could provide insight into evolutionary relationships and reconstructed evolutionary history using parsimony. While the former group made important methodological contributions, the rigorous logic and a solid intellectual basis of the latter prevailed. Modern systematics remains grounded on this basis, with perhaps one exception. The notion that we cannot sufficiently understand the evolutionary process to develop a model of it has given way to at least a more nuanced view, if not outright acceptance of model-based approaches. Certainly, the latter are preferred in evaluating the sequence data that form the basis of modern phylogenies. In parallel, rigorous quantitative approaches to comparative biology have also been developed [4,32,33].

**Figure 2:**
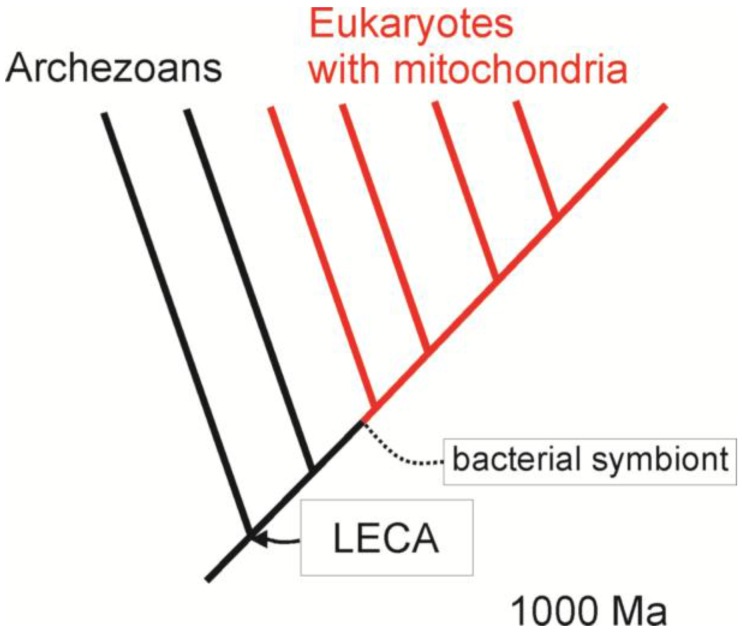
The archezoan hypothesis suggests that the last eukaryotic common ancestor (LECA) lacked mitochondria and had a relatively recent occurrence (<1000 Ma). By this view, several primitively amitochondriate eukaryotic lineages (in black) persist to the present along with mitochondriate lineages (in red).

For many years, the early evolution of eukaryotes seemed to fit comfortably into this framework. Eukaryotes were thought to have evolved and diversified to a limited extent until one lineage formed an endosymbiosis that led to modern, mitochondriate taxa (Figure 2). This archezoan hypothesis [34] provided an opportunity for the application of comparative methods. Because primitively amitochondriate eukaryotes were thought to persist to the present, a common ancestor of eukaryotes could be reconstructed with some of the derived features of modern eukaryotes. Combined with a phylogeny of mitochondriate eukaryotes, it would have been possible to discern which features were associated with the acquisition of mitochondria and which were not.

**Figure 3:**
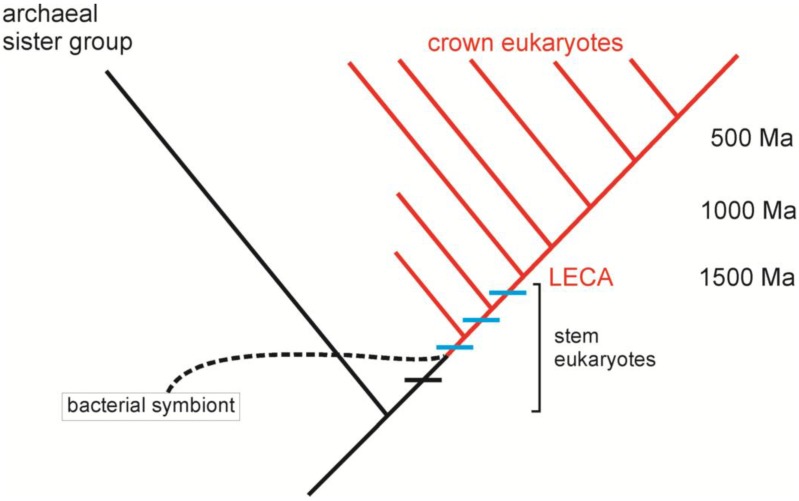
More recent data suggest that all derived features of modern eukaryotes, including mitochondria, were shared by the last eukaryotic common ancestor (LECA), which occurred >1500 Ma. The timing of the derivation of shared characters of eukaryotes (horizontal bars in stem eukaryotes) relative to the endosymbiosis that led to mitochondria (dashed line), remains unexplored. Did the derivation of these characters precede (black bar) or follow (blue bars) the endosymbiosis? (The chronological time scale for the stem eukaryotes is expanded for emphasis.)

Eventually as more became known, the archezoan hypothesis had to be abandoned. The putative amitochondriate eukaryotes were not sister-taxa to the mitochondrion-containing eukaryotes, nor did they truly lack mitochondria. Indeed, all eukaryotes were eventually found to have at least vestigial mitochondria (mitosomes and hydrogenosomes) [35]. Comparative genomics suggest that the common ancestor of all extant eukaryotes exhibited virtually all of the derived characters of the group [36]. Molecular phylogenetics and the comparative method are thus ineffective for inferring the process of eukaryogenesis. Because this process occurred in the eukaryotic stem group (for which all representatives are extinct), examining modern taxa remains uninformative in this context (Figure 3). Thus some of the most powerful tools of the modern synthesis can shed no light on one of the premier questions in the study of the history of life. Corroborative statements abound in the literature. For instance, Guy *et al.* point out: “The absence of such missing links, or intermediate stages of eukaryogenesis, significantly hampers the delineation of more sophisticated models for the emergence of the eukaryotic cell” [37]. Pittis and Gabaldón write: “The origin of eukaryotes stands as a major conundrum in biology. Current evidence indicates that the last eukaryotic common ancestor already possessed many eukaryotic hallmarks, including complex subcellular organization. In addition, the lack of evolutionary intermediates challenges the elucidation of the relative order of emergence of eukaryotic traits” [38].

## 4. Building a Framework to Explore the Origin of Eukaryotes

Several outstanding questions thus remain [39]; in particular, how and when were mitochondria acquired relative to the defining features of eukaryotes? To the extent that eukaryogenesis involved cycles of conflict and cooperation and that eukaryotic features can be shown to be sequelae of the endosymbiosis, progress can be made in answering these questions as suggested by the examples below. 

### 4.1. Introns and Endosymbiosis: From Small Things, Big Things One Day Come 

Cosmides and Tooby [21] highlight the possibilities of genomic conflict inherent in the mitochondrial endosymbiosis. Martin and Koonin [40] substantially advance and elaborate these themes. As endosymbionts died and released their DNA into the cytosol, symbiont DNA integrated into the host genome. Recombination and association with host promoters resulted in expression of symbiont genes in the cytosol [41]. This chimeric, proto-nuclear genome blurred the distinction between the host and the endosymbionts (now proto-mitochondria) and led to the emergence of the higher-level unit, the proto-eukaryote, encompassing both. The chimeric genome in turn provided opportunities for mobile genetic elements including group II introns that may have been present in the genome of proto-mitochondria. These introns spread throughout the proto-nuclear genome. Nevertheless, the slow rate of intronic splicing relative to translation led to serious problems with gene expression. A simple solution to this problem is a dedicated translation compartment, separate from that of transcription, *i.e.*, a physical barrier producing cytosol and nucleus.

As often happens in biological systems, a solution to one problem results in a cascade of sequelae. As developed by Martin *et al.* [42] and Garg and Martin [43], compartmentalizing chromosomes in a nucleus required that they no longer attach to the plasma membrane. Hence, when the cell divided, the chromosomes no longer automatically segregated. As a consequence, the proto-eukaryote may have grown to an enormous size by prokaryotic standards. Nevertheless, once surface-to-volume constraints at the new larger size became limiting, the need to successfully divide and segregate chromosomes would become acute. Garg and Martin [43] suggest that in the cytosol, which was rich with ATP supplied by the mitochondria, high levels of protein expression and experimentation occurred. Perhaps coupled with the newly derived large size, this may have led to microtubule dependent chromosome segregation. Ultimately, this process led to meiosis, the eukaryotic cell cycle, and mitosis [43].

An important point to recognize is the contingent nature of these events. Mitochondrial symbiosis necessarily preceded the intron proliferation, which led to the nucleus and the new difficulties with cell division that were solved by microtubule dependent chromosome segregation and the origin of the cell cycle. A clear sequence for these events can thus be discerned.

### 4.2. Conflict, Cooperation, and the Evolution of Eukaryotic Bioenergetics 

As mentioned repeatedly by Garg and Martin [43] among others, the features that permit meiosis and mitosis required a considerable supply of ATP, and this required mitochondria [44]. While a relatively seamless energetic coupling between the cytosol and mitochondria was thus necessary for many of the shared derived features of eukaryotes, achieving this coupling was anything but straightforward. In evolutionary terms, the problem is exactly as presented in Section 2: in nascent groups, cooperation is not automatic and an evolutionary advantage can often accrue to selfish defectors that favour their own replication at the expense of the good of the group. In the case of proto-mitochondria, the issue can largely be distilled down to bioenergetics: if releasing ATP into the cytosol diminishes the fitness of an individual endosymbiont, how did this evolve?

It is worth considering this question for a moment. The parallels to the general debate concerning individual and group selection are clear. During the initial metabolic symbiosis that gave rise to the eukaryotic cell, stoichiometry enforced symbiosis [27]. Once proto-mitochondria began to supply ATP to the cytosol, however, the opportunities for defection vastly increased. An endosymbiont that releases ATP into the cytosol will lower its own replication rate compared to one that does not, simply because on average more energy results in more replication. However, a group of endosymbionts within a proto-eukaryote will have a higher fitness if they all release ATP into the cytosol, because the proto-eukaryote will replicate faster when provisioned with abundant energy, and ultimately the replication of the endosymbionts depends on the replication of the proto-eukaryote. The evolutionary context is thus precisely the same as that which led to the debate in the 1960s [9,10,11]. What is favoured by selection at the level of the group is not favoured by selection at the level of the individual. All of the criticisms of Wynne Edwards [9] would apply with equal force to the idea that the endosymbionts would naturally cooperate for the good of the group. Indeed, even if a group of cooperative endosymbionts managed to evolve within a single proto-eukaryote, such a group would remain vulnerable to defection. If an individual endosymbiont ceased to release ATP and instead used this ATP for its own selfish replication, its descendants would out reproduce the co-operators. As a consequence, the defectors, which do not release ATP, would come to predominate, even if this led to the eventual demise of the proto-eukaryote.

The apparent difficulties with the evolution of eukaryotic bioenergetics are matched only by the crucial role that these energetics play in the evolution of eukaryotic complexity. Much of this complexity depends directly or indirectly on maintaining large size, a large nuclear genome, and high levels of both gene expression and protein-protein interactions. These features require a rich supply of ATP from mitochondria, and many analyses of eukaryogenesis simply assume that an abundant ATP supply was available in the cytosol from the moment the endosymbiosis began [35,43,44].

Mitochondrial bioenergetics provides a solution to this conundrum [27,28,29,30,31]. Mitochondria convert energy by oxidizing substrate and using the resulting reducing power in their electron transport chains to form a trans-membrane proton gradient. When protons return to the mitochondrial matrix through ATP synthase, they catalyze the formation of ATP from ADP and inorganic phosphate. ATP is then exported to the cytolsol via ADP/ATP carriers (AACs) that swap ATP for ADP. The electron transport chains of mitochondria function well when metabolic demand matches substrate oxidation. When metabolic demand falters, however, and substrate is still available, the electron carriers become highly reduced and formation of reactive oxygen species (ROS) increases dramatically. At high levels, ROS can damage various living systems. For endosymbionts inhabiting the novel environment *in hospite*, *i.e.*, in the host, many energy-demanding activities depend in turn on the activities of the host. For instance, if the host grows and divides, the endosymbionts can do so as well. On the other hand, if growth and division of the host ceases, metabolic demand in the endosymbionts likewise plummets. Under these circumstances, the problem for the endosymbionts would not be too little ATP, but rather too much. AACs could thus have evolved not as a mechanism to supply the cytosol with ATP, but as a mechanism to supply the proto-mitochondrial endosymbionts with ADP. AACs belong to a well-defined gene family that includes phosphate carriers and uncouplers [45]. The former provide the necessary complement to ADP, while the latter provide an alternative mechanism to accomplish the same result—uncouplers directly diminish the trans-membrane proton gradient and shift the redox state of the electron carriers toward oxidation.

If AACs and related carriers evolved in this manner, the problems of conflict and cooperation in eukaryotic bioenergetics are considerably simplified. First, if AACs evolved from proto-nuclear genes, installation of these carriers in the inner membrane of the proto-mitochondria would require the prior evolution of the protein import apparatus. Invention would thus be piled on invention. On the other hand, if AACs evolved from proto-mitochondrial genes, the protein import apparatus would not be required. Second, if AACs evolved as a straightforward adaptation of endosymbionts to the vicissitudes of their existence *in hospite*, the uncertain and in any event slower process of group selection need not be invoked. Rather, an individual proto-mitochondrion that evolved ACCs would be favoured by individual selection and its descendents would proliferate within a proto-eukaryote. That proto-eukaryote in turn would have an energetic boost to its own replication and would increase in the population. AACs and other carriers could also spread by lateral gene transfer, which would tend to homogenize the genetic complements of proto-mitochondria both within and between proto-eukaryotes.

It is worth reiterating that virtually all shared derived characters of eukaryotes depend directly or indirectly on the metabolic homeostasis manifest in modern representatives. Large size, a large nuclear genome, and high levels of both gene expression and protein-protein interactions depend on a steady supply of ATP from mitochondria and thus on AACs. The evolution of this crucial device must have preceded the origin of virtually all eukaryotic complexity. Since AACs required invention, however, mechanisms to maintain metabolic homeostasis that only required co-option likely evolved first [29,30].

### 4.3. Oxygen, Metabolism, Membranes, and Sex

Since atmospheric oxygen levels have shown dramatic changes over geological time, these levels have no doubt influenced a number of major events in the history of life. Nevertheless, based on the relatively rough correlations available, assertions of close functional relationships between oxygen and any particular events should be made with caution [46]. A case in point is the origin of the eukaryotes, which occurred perhaps 1750 Ma [47,48], and which followed the “Great Oxidation Event,” (GOE) the initial rise in atmospheric oxygen 2400 Ma [48]. Despite their metabolic simplicity as compared to prokaryotes, eukaryotes nevertheless have significant capabilities for anaerobic energy metabolism [49]. While eukaryotes produce sterols, these can be formed at minute concentrations of O_2_ [50]. Together, these findings [49,50] might suggest that aerobic metabolism was not characteristic of stem eukaryotes and LECA. The role of aerobic respiration and ROS formation as a selective agent in conflict mediation during the origin of eukaryotic metabolism may thus be called into question.

Recent evidence [51], however, shows that aerobic respiration can occur with only trace amounts of oxygen, far below the “Pasteur Point.” In view of these data, sterol synthesis likely provides a marker for aerobic respiration, and both may have preceded the GOE. Indeed, under conditions where the terminal electron acceptor, O_2_, is limiting, the redox state of the carriers of the electron transport chains tend to be shifted toward reduction. Despite the low concentration of oxygen, ROS can thus still form in significant quantities. As suggested by these data, facultative aerobic respiration and sterols very likely were features of the eukaryotic stem group and LECA.

Oxygen and sterol synthesis may relate to one of the biggest mysteries surrounding eukaryotes: the origin of sex [31]. If the host was a phagocytic archaean [37], perhaps it also engaged in sex? Indeed, some modern archaeans appear to have eukaryote-like cell fusion and genetic recombination [52,53]. Wall-less mutants of bacteria easily fuse [54]. Many bacteria improve membrane function using hopanoids. Archaeans, however, lack hopanoids [55]. Could a phagocytic archaean or proto-eukaryote have relied on sterols to stabilize its membranes? Functionally similar to hopanoids, sterol content in membranes tends to provide rigidity while maintaining fluidity. A phagocytic proto-eukaryote also could easily engulf the bacterial symbionts that became mitochondria.

Sex, however, has additional ramifications for endosymbionts because cell fusion allows them to migrate between cells. This tends to weaken group-level selection on endosymbionts and favor defectors. Radzvilacicius and Blackstone [31] examine the countervailing selection pressures using simple mathematical models. On balance, the evidence suggests that the destabilizing effects of selection for defectors outweigh potential advantages [31]. While aerobic metabolism and sterol synthesis may have been derived early by stem eukaryotes, sex is more likely to have been derived later.

## 5. Discussion

While oversimplified views of evolutionary theory may place endosymbiosis outside the realm of the modern synthesis, a careful examination shows that cooperation can emerge from cycles of conflict and conflict mediation. Endosymbiosis fits well within the multi-level generalization of Darwin’s theory, and indeed Darwin himself pioneered such a generalization. A much more serious challenge to the framework of the modern synthesis is posed by the eukaryotic common ancestor emerging fully formed from its prokaryotic predecessors. Modern molecular phylogenies and the comparative method—both cornerstones of the modern synthesis—can provide little insight under these circumstances.

In this context, it should be noted that the diversity of eukaryotes remains poorly characterized. Differences among modern eukaryotes may yet be discovered, perhaps leading to alternative reconstructions of LECA, the last eukaryotic common ancestor. Putatively early diverging eukaryotes (e.g., excavates [56,57]) may yet show some undiscovered differences from other eukaryotes (the neozoans). For instance, multicellular representatives of the excavates are notoriously scarce compared to those found in neozoans. Not only are other eukaryotes more frequently multicellular, but increasing amounts of experimental evidence suggest that in many neozoans even unicellular forms can quickly transition to multicellularity [58]. Excavates may lack some of the shared derived characters of neozoans related to conflict mediation [27]. On this basis and perhaps others, reconstruction of LECA may yet be altered.

Similarly, a better understanding of the affinities of the components of the eukaryotic cell may provide additional insight. While the sister-group relationship between mitochondria and alphaproteobacteria has been clear for some time, the affinities of the host cell remain murky. Likely, the host nested within a large clade of archeans [37], with its closest living relative the newly discovered Lokiarchaeon [59]. Nevertheless, close sister groups of the host may presently remain undiscovered, or may possibly have succumbed to extinction.

At this point, however, the best tools for reconstructing eukaryogenesis in extinct stem groups are various lines of indirect evidence. For instance, Lynch and Marinov [60] suggest that in eukaryotes with small effective population sizes and thus high levels of genetic drift, increased genome size follows from increased cell size. By their view, the sequence of events of eukaryogenesis was increased cell size, then genomic complexity. It is arguable whether either of these events preceded the mitochondrial endosymbiosis. Because of the scaling of surface to volume, the energy conversion system of a prokaryote, which is bound to the external membrane, becomes increasingly inefficient as size increases [61]. One way to circumvent this constraint is to move small, energy-converting cells inside a larger cell. Once AACs evolve, the external membrane of the larger cell can cease to function in energy conversion. The sequence of events proposed by Lynch and Marinov would thus seem to be: mitochondria, larger size, and then genomic complexity.

Pittis and Gabaldón [38] reconstruct the sequence of eukaryogenesis by inferred genetic distances. While their approach may be compromised by methodological issues (e.g., unsampled sister taxa, as discussed above), they suggest that the oldest eukaryotic genes have affinities to archaeans, while the youngest have affinities to alphaproteobacteria. From these data, they suggest that much of the complexity of the eukaryotic cell developed prior to the mitochondrial symbiosis.

A number of studies are thus using various approaches to reconstruct the sequence of events of eukaryogenesis. The approach suggested here involves logical assumptions that are well founded on evolutionary theory. The multi-level theory predicts cycles of conflict and conflict mediation with the shared derived features of eukaryotes emerging as by-products [27,28,29,30]. A sequence of events can thus emerge. The geological time scale provides an apt analogy. A set of logical assumptions (e.g., older strata lie below younger, while inclusions are older still) allowed building this time scale. Here, a variety of logical assumptions have been brought to bear on the timing of events during eukaryogenesis. Some of these are more theoretical (e.g., individual selection tends to be stronger than group selection, co-option of existing structures occurs more rapidly than invention of new ones), while other are more empirical (e.g., introns originated in bacteria). All of these assumptions can and should be further examined.

**Figure 4:**
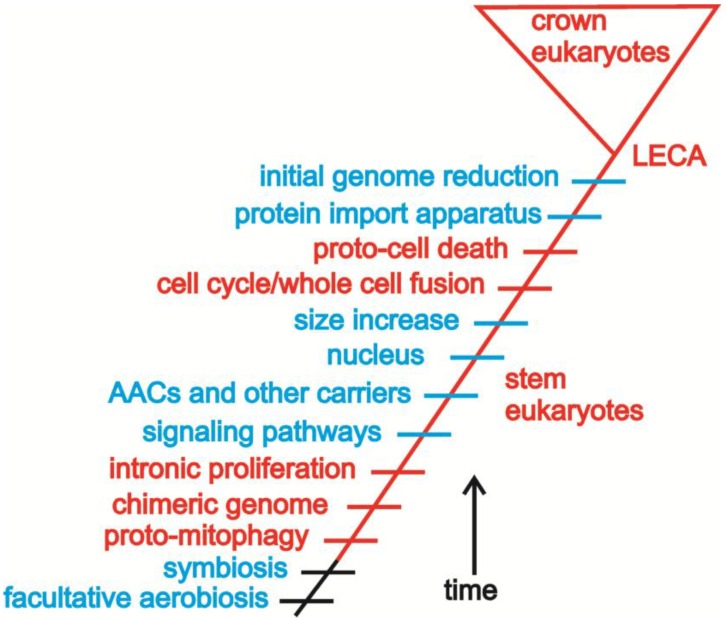
Based on logical assumptions, a sequence of events during eukaryogenesis is outlined. Derived characters that contribute more to conflict are highlighted in red, while those that mediate conflict are in blue. The list of characters is of course incomplete and provided only as an exemplar. (The chronological time scale for the stem eukaryotes is expanded for emphasis.)

The focus of this review, however, is not the validity of any of the assumptions. Rather, the focus is on the overall approach: if a set of logical assumptions can be developed, then the relative timing of events during eukaryogenesis can be understood. Each of the examples described in Section 4 suggests portions of the sequence of events (e.g., endosymbiosis led to intronic proliferation and the nucleus). The obvious difficulty involves combining these, and other, examples. Figure 4 outlines a possible approach to interlacing some of the events during eukaryogenesis. This is not meant to be a definitive analysis, but rather a proof of concept.

Sterol synthesis and a facultative aerobic metabolism may predate the symbiosis, which rapidly developed into an endosymbiosis. The stoichiometry of the metabolic complementation initially enforced cooperation [27]. Consumption of endosymbionts by the host (“proto-mitophagy” in Figure 4) likely began immediately, with obvious potential to destabilize the endosymbiosis [30]. Release of endosymbiont DNA led to a chimeric, proto-nuclear genome, rife with proliferating introns, again leading to conflict. Co-option of existing prokaryotic signaling pathways into new proto-eukaryotic roles may have allowed the proto-mitochondria to survive in the challenging environment within the proto-eukaryote [29,30]. The first inventions of the proto-eukaryote further mediated conflict. AACs and other carriers better facilitated metabolic homeostasis and as a by-product led to an ATP-rich cytosol. The nucleus alleviated conflict stemming from intronic proliferation, but also complicated successful cell division. Size increase may have occurred at this point if not earlier in the process of eukaryogenesis. Ultimately, evolution of the eukaryotic cell cycle rescued nucleus-containing proto-eukaryotes [43]. By allowing horizontal migration of proto-mitochondria, fusion introduced additional conflict stemming from the manipulation of the cell cycle by proto-mitochondria. The antecedents of programmed cell death evolved in this context [26,61,62]. Ultimately, the invention of the protein import apparatus allowed gene loss from the mitochondrial genome, a highly effective mechanism of conflict mediation [63]. Nevertheless, mitochondria retain some heritable variation, since for purposes of redox signaling some genes remain anchored there [64,65]. All of these events likely occurred rapidly over a geologically instantaneous period of time (note that the time scales for the stem eukaryotes in Figure 3 and Figure 4 are vastly expanded for emphasis).

## 6. Conclusions

Second only to the origin of life, the origin of eukaryotes remains biology’s greatest mystery, seemingly at odds with the modern synthesis. A careful analysis, however, shows that the eukaryotic endosymbiosis indeed fits well within the multi-level theory of evolution. On the other hand, the lack of any intermediates between modern prokaryotes and eukaryotes stymies comparative and phylogenetic methods. A series of logical assumptions based on evolutionary theory, however, can provide a framework for understanding eukaryogenesis. Cycles of conflict and conflict mediation likely produced many of the shared derived characters of eukaryotes. An analysis of the relative timing of these events suggests that endosymbiosis preceded and indeed precipitated the evolution of many of the features that define eukaryotes. The modern synthesis thus provides sufficient scope to develop an evolutionary framework to understand the origin of eukaryotes.
